# Exploring spatial distribution of social vulnerability and its relationship with the Coronavirus disease 2019: the Capital region of South Korea

**DOI:** 10.1186/s12889-022-14212-7

**Published:** 2022-10-10

**Authors:** Donghyun Kim

**Affiliations:** Department of Urban Planning and Engineering, Pusan National University, 2, Busandaehak-ro 63beon-gil, Geumjeong-Gu, Busan, 46241 Korea

**Keywords:** Spatial distribution, Social vulnerability, Coronavirus disease 2019, South Korea

## Abstract

**Background & objective:**

The ongoing coronavirus disease 2019 (COVID-19) pandemic continues to cause death and socioeconomic problems worldwide. This study examined the spatial distribution of social vulnerability to COVID-19 and its relationship with the number of confirmed COVID-19 cases in 2020, focusing on the Capital region of South Korea.

**Methods:**

A traditional social vulnerability index (SVI), healthy SVI, and the difference of each SVI were constructed in 2015 and 2019. The traditional SVI was constructed across five domains: age, socioeconomic disadvantage, housing, income, and environment. The healthy SVI domains were: prevention, health-related habits, chronic disease, healthcare infrastructure, and mortality. The spatial distribution of the traditional SVI, healthy SVI, and confirmed cases of COVID-19 was explored using ArcGIS 10.5. Pearson correlation was used to identify the relationship between confirmed COVID-19 cases and the two SVIs and their changes between 2015 and 2019. Four multiple linear regression models were used to identify the impact of the changes of the two SVIs on the confirmed COVID-19 cases for the three episodes and total period with control of population using STATA/MP 16.1.

**Results:**

Confirmed COVID-19 cases were concentrated in a specific area of the Capital region. The traditional SVI was more vulnerable in the outer regions of the Capital region, and some central, western, and eastern areas reflected an increase in vulnerability. Healthy SVI was more vulnerable in the northern part of the Capital region, and increase in vulnerability showed in some central areas above Seoul. By multiple regression with the population controlled, the difference of the traditional SVI between 2015 and 2019 showed a positive relationship with the confirmed COVID-19 cases in all models at a significance level of 0.05, and the 2019 integrated SVI showed a negative relationship with confirmed COVID-19 cases in all models.

**Conclusions:**

The results of this study showed that the confirmed COVID-19 cases are associated with increased traditional SVI vulnerability between 2015 and 2019 and have a high positive relationship with the spread of COVID-19. Policy efforts are needed to reduce confirmed COVID-19 cases among the vulnerable in regions with relatively increased traditional SVI.

**Supplementary Information:**

The online version contains supplementary material available at 10.1186/s12889-022-14212-7.

## Introduction

Since a cluster of pneumonia cases of unknown etiology was discovered in Wuhan City in December 2019, the coronavirus disease 2019 (COVID-19) has caused deaths and socioeconomic problems worldwide [1, 2]. As of December 3, 2021, there have been 264,099,245 total cases of COVID-19 and 5,233,277 related deaths worldwide; in Korea specifically, there have been 457,612 total cases and 3,705 related deaths [3]. Following the impact of COVID-19 on public health and the economy [4], social vulnerability and spatial heterogeneity have been reported to be the potential socioeconomic determinants of COVID-19.

Social vulnerability is commonly discussed in the context of disasters [5, 6]. Vulnerability is a key concept of precaution; it is a pathway through which society can predict and resist the impact of an event and return to the pre-event state with potential losses [7, 8]. The concept of social vulnerability can be defined as social groups’ susceptibility and resilience toward the impacts of disaster [9]. Social vulnerability focuses on social variables such as socioeconomic status, education level, and race to understand response to external disasters [8], and is based on the social risk factors of external disasters [5, 10]. Social risk factors such as lack of access to resources, low political power, social capital, age, building stock, beliefs and customs, physically limited individuals, and infrastructure were discussed [11]. From a spatial perspective, vulnerability is not a mere risk factor of disaster but a socioeconomic phenomenon that serves as a criterion for assessing the pre-disaster state [12, 13].

Impacts of a disaster are not evenly distributed across affected regions [14]. Socioeconomic conditions and resources are essential for disaster response [15]; they determine a region’s responsiveness to a disaster and the speed at which normalcy can be restored. COVID-19 also exhibits spatial heterogeneity, which is associated with factors of social vulnerability, such as age, accessibility to public healthcare, and the socioeconomic index [16].

Recent studies on social vulnerability and COVID-19 regard the pandemic as a disaster [15]. Studies have reported that low-income earners are more susceptible to the negative effects of COVID-19 [17–19], as such people tend to display poor adherence to preventive measures—such as social distancing—due to their economic activities and housing conditions. Additionally, they are at an increased risk of infection due to poor environmental sanitation levels and have low access to public healthcare services. Those with socioeconomic disadvantages, ethnic groups (e.g., blacks and Hispanics), and older adults are the most vulnerable during global pandemics [20–25]. Social vulnerability is an important contributor exacerbating a group’s risk during a disaster [26, 27]. Existing studies on social vulnerability discuss various factors that impact the same, such as income, education, nutrition, transportation, housing, employment, stress, and healthcare [28–30]. Studies on the relationship between COVID-19 and social vulnerability have reported that the older adults, low-income populations with low education levels, and ethnic minorities experience a greater burden due to COVID-19 [15, 23, 31–33].

COVID-19 spreads through respiratory droplets or direct contact [34]. This implies that the spatial range of geographical movements and socioeconomic activities plays an important role in the spread of the virus [35, 36]. Previous studies have examined the relationship between spatial heterogeneity of the social vulnerability index (SVI) and COVID-19 at the regional level within a nation [16, 31, 33, 37] or the community level within a city [23, 38]. Most previous studies that were based on or revised the CDC’s data and concept or revised, suggested a positive relationship between COVID-19 and SVI [15, 16, 19, 23, 32, 33, 38, 39]. However, some studies discussed the inconsistent relationship between COVID-19 and SVI by the phase of episode and spatial context regardless of same spatial scope [31, 40, 41]. These studies had the common limitations that changes in SVI were not considered and they did not consider the different characteristics of SVI, which can be divided into socioeconomic factors (traditional SVI) and health factors (healthy SVI). The research questions that prompted these studies were: why does the occurrence of COVID-19 differ despite the spatial scope of areas with similar socioeconomic characteristics? How is this difference related to the spatial heterogeneity of social vulnerability?

This study aimed to assess the spatial distribution of social vulnerability in the Capital region of South Korea and investigate its relationship with the number of confirmed COVID-19 cases. First, social vulnerability was measured using the traditional SVI with five domains (age, socioeconomic disadvantage, housing, income, and environment) and the healthy SVI with five domains (prevention, health-related habits, chronic disease, healthcare infrastructure, and mortality). Next the spatial distribution of SVI and the difference of SVI was assessed by each domain using five quantile maps for 2015 and 2019. Finally, the relationship between the number of confirmed COVID-19 cases and the differences in the traditional and healthy SVIs from 2015 to 2019 were investigated using multiple linear regression.

The Capital region of South Korea comprises the Seoul Metropolitan Area, the Gyeonggi-do, and the Incheon Metropolitan Area. These areas were selected as study sites as they have relatively similar socioeconomic characteristics as compared to non-Capital regions. For instance, all three areas have high population density and relatively small surface areas in which all economic activities are concentrated. Although the surface area of the Capital region only accounts for 11.8% of the country’s total land area, approximately half (50.3%) of the Korean population resides there and contributes to 46.8% of the national economic output. The Capital region contained 62% of all confirmed COVID-19 cases in 2020. Thus, the current study focused on the Capital region as one regional unit of economic function, which shares common characteristics of economic activities, such as commuting, labor market, and consumption. However, the socioeconomic conditions in one regional unit may be different from the specific spatial unit. Considering the spatial heterogeneity of living conditions and the vulnerability status of persons living in the Capital region [42, 43], the region was deemed suitable for assessing the spatial heterogeneity of social vulnerability and its relationship with the number of confirmed COVID-19 cases. The units of analysis were the local government units (si, gun, and gu).

## Literature review

The impact of COVID-19 on socially vulnerable populations is unevenly distributed [32]. Social inequality in traditionally vulnerable populations can exacerbate the negative impacts [44] of the pandemic and favor the spread of the virus [17]. Recent studies have reported that social determinants contribute to the heterogeneous risks of COVID-19 among different population groups, and that people in socially vulnerable regions are at a very high risk of contracting COVID-19 [21–24, 45, 46].

Social vulnerability is associated with the number of confirmed COVID-19 cases and deaths. Karaye and Horney [33] suggested that there is a relationship between SVI and the rate of increase in the cumulative number of confirmed COVID-19 cases. Khazanchi et al. [47] found that the infection rate and mortality due to the disease were higher in socially vulnerable countries. Similarly, Nayak et al. [48] observed a relationship between SVI and COVID-19 mortality. Wang et al. [32] reported spatial variations in the relationships between social vulnerability and the number of confirmed COVID-19 cases and deaths. In their longitudinal analysis, Neelon et al. [31] confirmed the spatial heterogeneity in the relationship between social vulnerability and number of confirmed COVID-19 cases at the county level.

The following factors are considered in SVI: socioeconomic, housing composition, minority status and language, and accessibility to housing and transportation [37, 49, 50]. The Centers for Disease Control and Prevention in the United States (US CDC) created an SVI considering the external stresses on human health [51].

These SVIs have been used in the previous studies on COVID-19 and SVI. Wang et al. [32] assessed the relationship between social vulnerability factors and COVID-19 at the county level in the United States. They included confirmed COVID-19 cases and deaths as of July 21, 2020, and used data from the 2018 American Community Survey to obtain social vulnerability factors. Using a percentile ranking method, social vulnerability was determined using 15 variables that were divided into four groups: socioeconomic factors, housing composition and disability, minority status and language, and housing and transportation. The relationship between social vulnerability and COVID-19 was then assessed. Surgo Ventures [52] developed the COVID-19 Community Vulnerability Index using SVI data from the US CDC. Infectious factors, healthcare systems, high-risk environments, and population density were added to the variables that comprised the existing SVI. They reported an association between the characteristics of socially vulnerable groups and COVID-19 deaths at the county level and the impact of spatial heterogeneity on COVID-19.

Tiwari et al. [37] used machine learning and a non-linear algorithm to develop an SVI. They examined the relationship between COVID-19 and the SVI at the county level using data of confirmed COVID-19 cases and deaths as of July 31, 2020, in the United States. They developed an SVI by combining the index developed by Flanagan et al. [10] with the infection and healthcare system factors used by Surgo Ventures [52] and found spatial heterogeneity in the association between social vulnerability and COVID-19.

Snyder and Parks [15] used factors of COVID-19 variables classified into four groups (ecological, social, health, and economic factors) to create an SVI. They also examined vulnerability within the United States at the county level. The variables used were potential major indicators of infectious disease; therefore, the association of each variable with COVID-19 was assessed. De Souza et al. [19] examined the relationship between COVID-19 and a 49-variable SVI using data of confirmed COVID-19 cases and deaths as of May 6, 2020, in Brazil. They determined the incidence, mortality, and case fatality of COVID-19, and identified the demographic and social determinants of social vulnerability using an exploratory analysis, spatial clustering, and regression analysis.

Singh [53] conducted a survey in India between March 2020 and April 2020 to investigate social vulnerability to COVID-19 at a household level and to examine the sensitivity and adaptive capacity of five rural communities. Exposure levels were calculated using awareness of COVID-19, the quarantine period, and testing procedures during one’s experience with health hazards in the last five years. Sensitivity was based on living conditions, whereas adaptive capacity was calculated using income and health insurance data. Singh found that regions with low adaptive capacities had a high sensitivity [53]. Neelon et al. [31] used US CDC data [51] to determine social vulnerability and examine its relationship with the number of confirmed COVID-19 cases and deaths. They developed an SVI that was similar to those developed by Flanagan et al. [10] and Wang et al. [32], and used the national average for counties with unavailable data. The percentage of female residents, smokers, primary care physicians, average temperature, precipitation, population density, PM 2.5, and the proportion tested for COVID-19 were added to their SVI.

To create an SVI and compare the relationship between it and COVID-19 mortality between African Americans and non-African Americans, Kim and Bostwick [23] used responses from 800 census tracts within 77 communities of Chicago. The SVI was calculated using the US CDC’s method, and a principal component analysis was employed. Additionally, a health risk index was created using data from cases with chronic diseases. Karaye and Horney [33] created an SVI at the county level and performed a spatial regression analysis using socioeconomic variables. They examined the relationship between SVI and confirmed COVID-19 cases and identified several regions with a strong association between the two. Coelho et al. [16] examined the spatial heterogeneity in the spread of COVID-19 in Brazil using various social vulnerability indices, which were created using demographic factors, age, accessibility to public healthcare, and socioeconomic status. Biggs et al. [38] analyzed the relationship between social vulnerability and COVID-19 within the census tracts of Louisiana using the SVI developed by the US CDC.

These studies investigating SVI and COVID-19 showed different findings of the relationship between COVID-19 and SVI. Some studies suggested positive relationships between the confirmed cases or incidence rate of COVID-19 and SVI [14, 17, 21, 30, 31, 36, 39], but some studies showed different findings of their relationship, which varied by the periods of episode [31] and specific spatial context, such as rural areas [40]. Some studies suggested an inconsistent relationship between the incidence of COVID-19 and SVI [41].

## Methods

### Variables and data

In this study, the traditional SVI and healthy SVI are illustrated in Table 1. The traditional SVI comprised variables commonly used in disaster studies, whereas the healthy SVI comprised infection-related variables describing COVID-19 characteristics. Nine variables from five domains (age, socioeconomic disadvantage, housing, income, and environment) made up the traditional SVI. Eleven variables from five domains (prevention, health-related habits, disease, healthcare infrastructure, and mortality) made up the healthy SVI. These SVIs were created using data from the pre-COVID-19 period of Korea: 2015 and 2019. The number of confirmed cases per 10,000 persons was calculated by using the number of confirmed domestic cases registered daily from the second week of January 2020 to December 2020 by the Korea Disease Control and Prevention Agency and local governments, and the total population in 2020.

**Table 1:** Domain, variables, definition, and sources of SVI

	Domain	Variable	Definition	Data Source	Notes	References
Traditional SVI	A. Age	Older population	Percentage of older adults aged ≥ 65 years among the total registered resident population	Resident Population Statistics—Ministry of the Interior and Safety		Snyder and Parks (2020) [15], Neelon et al. (2021) [31], Biggs et al. (2021) [38], Kim and Bostwick (2020) [23], Islam et al. [41]
	B. Socioeconomic disadvantage	Foreign minorities	Percentage of foreigners not of Korean descent or from an OECD country among the total foreigner population	Foreigner Arrival and Departure Statistics—Ministry of Justice		Snyder and Parks (2020) [15], Tiwari et al. (2021) [37], Cohen-Cline et al. (2021) [49]
		Vulnerable groups	Percentage of low-income households, social assistance recipient households, and one-person households with an elderly social assistance recipient among the total number of households	Regional Statistics—Statistics Korea		Snyder and Parks (2020) [15], Neelon et al. (2021) [31], Biggs et al. (2021) [38], Amram et al. (2020) [39], Tiwari et al. (2021) [37], Islam et al. [41]
		Disabled persons	Percentage of disabled individuals among the total population	Disability Statistics—Ministry of Health and Welfare		Biggs et al. (2021) [38], Islam et al. [41]
	C. Housing	Old houses	Percentage of selected houses older than 30 years	Housing Census—Statistics Korea		Biggs et al. (2021) [38]
	D. Income	Pension income	Average national pension amount per beneficiary	National Tax Statistics—National Pension Service	Reverse direction	Snyder and Parks (2020) [15], Biggs et al. (2021) [38], Islam et al. (2021) [41], Khan et al. (2022) [40]
		Earned income	Average earned income reported to the National Tax Service per person	National Tax Statistics National Tax Service	Reverse direction	Snyder and Parks (2020) [15], Biggs et al. (2021) [38], Islam et al. (2021) [41]
	E. Environment	Particulate Matter (PM) 2.5	Annual average concentration (For measurements obtained from multiple locations, the average for all locations is used)	Air Pollution Status—Ministry of Environment		Snyder and Parks (2020) [15], Neelon et al. (2021) [31], Khan et al. (2022) [40]
		Particulate Matter (PM)10	Annual average concentration (For measurements obtained from multiple locations, the average for all locations is used)	Air Pollution Status—Ministry of Environment		Snyder and Parks (2020) [15]
Healthy SVI	F. Prevention	Rate of engaging in physical activities	Percentage of individuals who engage in intense physical activities for at least 20 min/day for at least three days per week or moderate physical activities for at least 30 min/day for at least five days in the last one week	National Health Screening Statistics - National Health Insurance Service	Reverse direction	Neelon et al. (2021) [31], Khan et al. (2022) [40]
		Influenza immunization rate	Percentage of individuals vaccinated against the influenza virus (flu) in the past year	Community Health Survey—Korea Disease Control and Prevention Agency	Reverse direction	Tiwari et al. (2021) [37]
	G. Health-related habits	Smoking	Percentage of current smokers who have smoked at least five cartons of cigarettes throughout their lifetime	Community Health Survey—Korea Disease Control and Prevention Agency		Snyder and Parks (2020) [15], Neelon et al. (2021) [31], Kim and Bostwick (2020) [23], Khan et al. (2022) [40]
		Obesity	Percentage of individuals with a body mass index ≥ 25 kg/m^2^	Community Health Survey—Korea Disease Control and Prevention Agency		Snyder and Parks (2020) [15], Kim and Bostwick (2020) [23], DeCaprio et al. (2020) [54], Khan et al. (2022) [40]
	H. Chronic disease	Hypertension	Percentage of individuals aged ≥ 30 years diagnosed with hypertension	Community Health Survey—Korea Disease Control and Prevention Agency		Snyder and Parks (2020) [15], Kim and Bostwick (2020) [23], DeCaprio et al. (2020) [54]
		Diabetes	Percentage of individuals aged ≥ 30 years diagnosed with diabetes	Community Health Survey—Korea Disease Control and Prevention Agency		Snyder and Parks (2020) [15], Kim and Bostwick (2020) [23], DeCaprio et al. (2020) [54]
	I. Healthcare infrastructures	Number of medical facilities	Number of health care facilities per 10,000 persons	Health Insurance Statistics—National Health Insurance Service Health Insurance Review and Assessment	Reverse direction	Tiwari et al. (2021) [37], Kim and Bostwick (2020) [23]
		Number of beds	Number of beds per 10,000 persons	Health Insurance Statistics—National Health Insurance Service Health Insurance Review and Assessment	Reverse direction	Snyder and Parks (2020) [15], Coelho et al. (2020) [16], Khan et al. (2022) [40]
		Number of professionals	Number of professionals per 10,000 persons	Health Insurance Statistics—National Health Insurance Service· Health Insurance Review and Assessment	Reverse direction	Tiwari et al. (2021) [37]
	J. Mortality	Mortality of respiratory diseases	Percentage of deaths from respiratory diseases per 100,000 persons (J00–J98,U04)	Cause of Death Statistics—Statistics Korea		Amram et al. (2020) [39]
		Mortality of infectious and parasitic diseases	Percentage of deaths from certain infections and parasitic diseases (A00–B99) per 100,000 persons	Cause of Death Statistics—Statistics Korea		Tiwari et al. (2021) [37]

*Note: OECD* Organization for Economic Co-operation and Development

The traditional and healthy SVIs were calculated by summing the scores of all the variables of the indices [15, 38, 50]. An integrated SVI, which is a combination of traditional and healthy SVIs, was also calculated. The SVI score for each domain was evaluated using the method developed by Flanagan et al. [44], Snyder and Parks [13], and Biggs et al. [36], in which a percentile rank was calculated for a spatial unit over all variables; the mean of the variables within each domain was also calculated [55]. The scores for all domains were summed to determine the final value of the traditional and healthy SVIs [38]. The scores for the traditional and healthy SVI were summed to obtain the integrated SVI. Due to the characteristics of percentile rank, the value of each variable of index and summed value from variables are consistent. The five quantile criterion, which were used by Snyder and Parks [15] and De Souza et al. [19], can be interpreted that first quantile (below 20%) constitutes low vulnerability and the fifth quantile (above 80%) indicates very high vulnerability. The difference of SVI was calculated subtracting the value of each SVI from 2015 from the SVI in 2019. If the percentile rank of SVI of a spatial unit is relatively increased in 2019 compared to 2015, the value of the difference is positive and vice versa. This study compares the traditional, healthy, and integrated SVI through the quantile maps of 2015 and 2019, as well as the spatial distribution of confirmed cases of COVID-19 per 10,000 in 2020 using a geographic information system (GIS).

### Statistical analysis

In Korea, three disease episodes of COVID-19 were identified: the first episode was from the Shinchenji church and Deanam hospital cases, the second was from the Gwanghawmun rally, and the third was from the community infection at the end of the year [56]. In this study, four models were constructed to understand the relationship between confirmed COVID-19 cases and the difference of traditional and healthy SVI within the three episodes, as well as all confirmed cases in 2020. Each model was divided by the confirmed COVID-19 cases in the episode period, which was dependent variable. Model 1 was used the confirmed cases from Weeks 8–11 (first episode), Model 2 used Weeks 33–37 (second episode), Model 3 used in Weeks 47–53, and Model 4 used all of 2020.

A multiple linear regression was performed for each model to investigate the relationship between the increase of the traditional and healthy SVI and the confirmed COVID-19 cases for the three episode periods and the total period when controlling for the 2019 SVI and population of 2020 (Eqs. 1, 2, 3 and 4). The number of confirmed COVID-19 cases during the first, second, and third episodes and the cumulative number of confirmed cases in 2020 were used as dependent variables. The differences between the 2015 and 2019 traditional and healthy SVIs were used as independent variables. Population in 2020, highly associated with confirmed COVID-19 cases, and the integrated SVI of 2019, which consists of the sum of the traditional and healthy SVI were used as independent variables. STATA/MP 16.1 software was used.

1 $$\mathrm{Model}\;1:\;{EP1COV}_i=\beta_0+{\beta_1DifftradSVI}_i+{\beta_2DiffhealSVI}_i+\beta_3{Pop}_i+\beta_4{IntSVI}_i+\varepsilon$$

2 $$\mathrm{Model}\;2:\;{EP2COV}_i=\beta_0+{\beta_1DifftradSVI}_i+{\beta_2DiffhealSVI}_i+\beta_3{Pop}_i+\beta_4{IntSVI}_i+\varepsilon$$

3 $$\mathrm{Model}\;3:\;{EP3COV}_i=\beta_0+{\beta_1DifftradSVI}_i+{\beta_2DiffhealSVI}_i+\beta_3{Pop}_i+\beta_4{IntSVI}_i+\varepsilon$$

4 $$\mathrm{Model}\;4:\;{TOTCOV}_i=\beta_0+{\beta_1DifftradSVI}_i+{\beta_2DiffhealSVI}_i+\beta_3{Pop}_i+\beta_4{IntSVI}_i+\varepsilon$$

where $i$ is the spatial unit (local administrative unit, *si-gun,gu*). *EP1COV, EP2COV,* and EP3COV are the numbers of confirmed COVID-19 cases in the first, second, and third episodes, respectively. *TOTPCOV* is the number of confirmed COVID-19 cases in 2020. *DifftradSVI* is the difference in the traditional SVI between 2015 and 2019. *DiffhealSVI* is the difference in the healthy SVI between 2015 and 2019. *Pop* is the population in 2020. *IntSVI* is the integrated SVI in 2019. $\varepsilon$ is the error term.

Considering the criteria for skewness and kurtosis proposed by West et al. [57] and Hong et al. [58], the dependent variables in Models 1–4 all satisfied the normality assumption. Further, Kolmogorov–Smirnov's normality test revealed that Models 2, 3, and 4 satisfied the normality assumption. In Model 1, the condition for normality was rejected as a result of the Kolmogorov–Smirnov's normality test, but the number of samples was sufficient to maintain the assumption of normality and perform multiple regression analysis. As for the variance inflation factor, multicollinerity was not detected. Regarding the White test for heteroscedasticity, Model 1 had the problem of heteroscedasticity, so it was estimated using robust standard error. The spatial correlation was not detected in all models.

## Results

### Spatial distribution of SVI in the Capital region

**Fig. 1:**
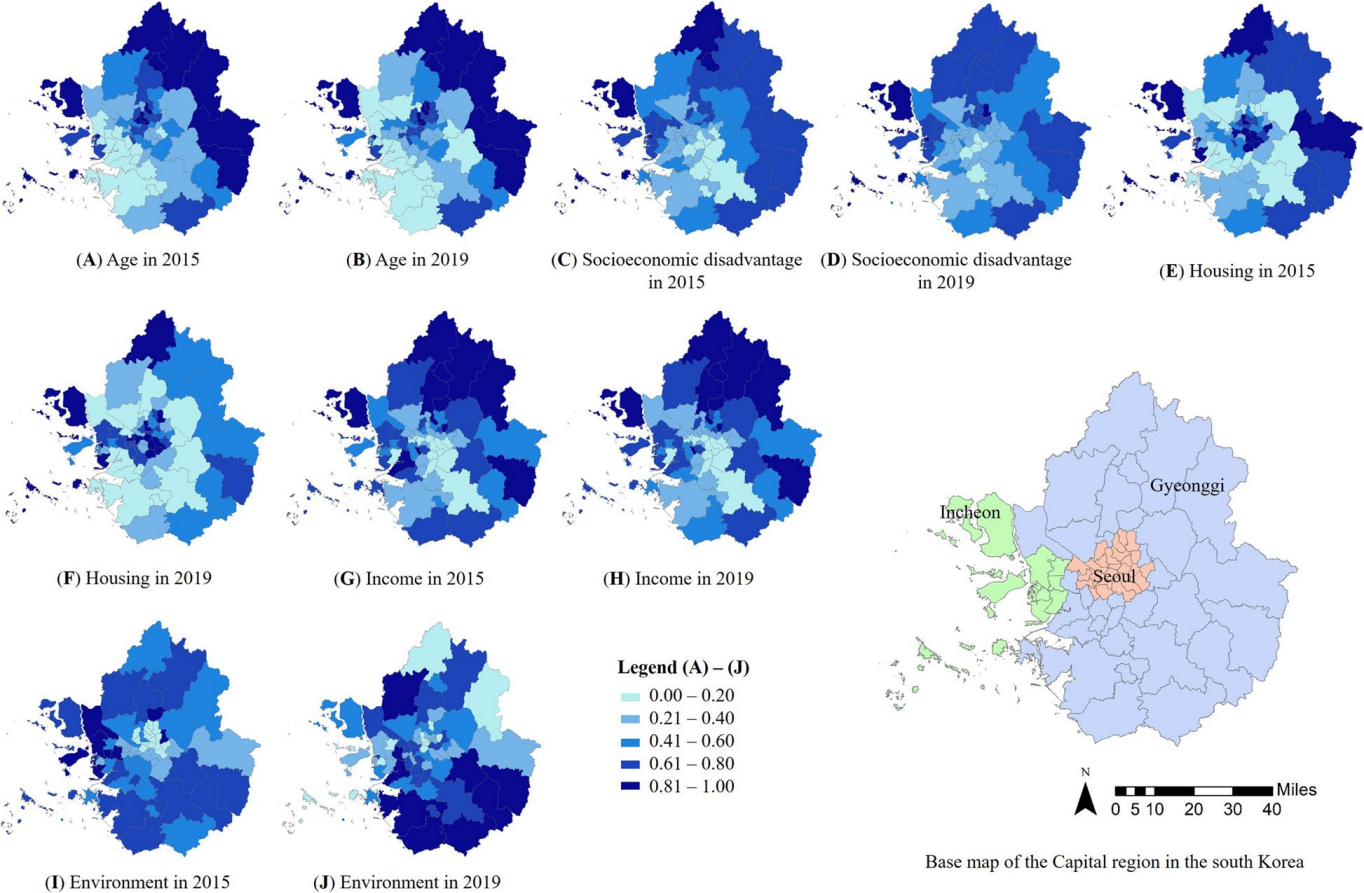
Quantile Map of domains in Traditional SVI

**Fig. 2:**
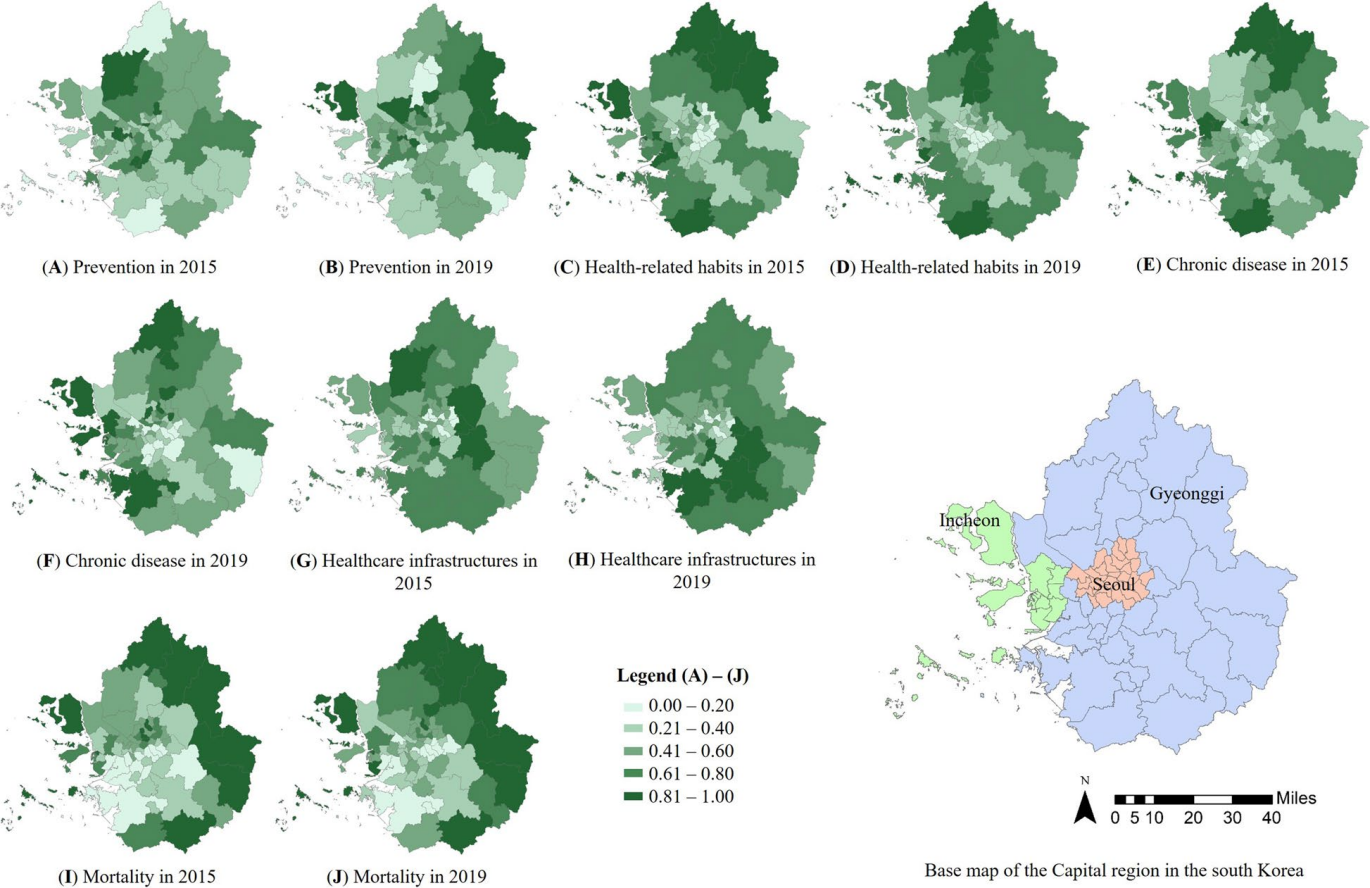
Quantile Map of domains in Healthy SVI

**Fig. 3:**
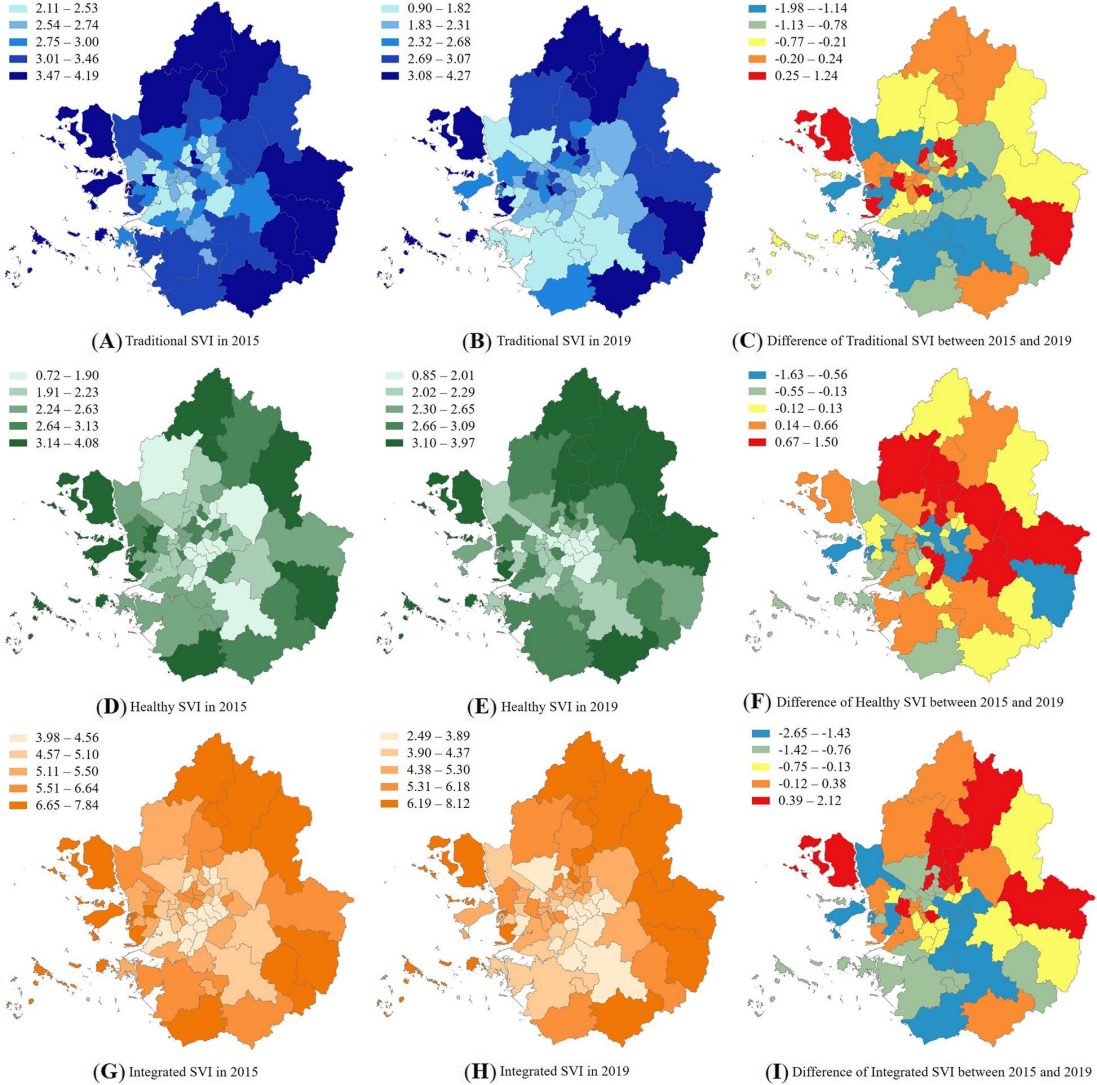
Quantile Map of traditional, healthy, integrated SVIs and difference of each SVI in 2015 and 2019

Figures 1, 2 and 3 show the distribution of SVIs in the Capital region. Figure 1 shows a quantile map of the indices for each domain of the traditional SVI. The regional distributions of the indices of the traditional SVI varied with the domain. Based on age (A domain index, Fig. 1 (A) and (B)), eastern Gyeonggi and northern Seoul were vulnerable. Based on socioeconomic disadvantage (B domain index, Fig. 1 (C) and (D)), northern Seoul, eastern, southern, and northern Gyeonggi, and western Incheon were vulnerable. Based on housing (C domain index, Fig. 1 (E) and (F)), the central region of Seoul, eastern Gyeonggi, and most of Incheon were vulnerable. Based on income (D domain index, Fig. 1 (G) and (H)), the south-central area of Seoul and Gyeonggi were not vulnerable, and northern, southern, and western Gyeonggi and Incheon were vulnerable. Based on the environment (E domain index, Fig. 1 (I) and (J)), the southern and northern regions were vulnerable.

Figure 2 shows a quantile map of the indices for each domain of the healthy SVI. The regional distributions of the indices of the healthy SVI varied with the domain. Based on prevention (F domain index, Fig. 2 (A) and (B)), some of the northern and eastern regions were vulnerable. Based on health-related habits (G domain, Fig. 2 (C) and (D)), south-central Seoul was not vulnerable but the suburbs were vulnerable. Based on chronic disease (H domain index, Fig. 2 (E) and (F)), some regions of the suburbs and northern Seoul were vulnerable. Based on the healthcare infrastructure (I domain, Fig. 2 (G) and (H)), most regions, except some of central Seoul, were vulnerable. Based on mortality (Fig. 2 (I) and (J)), the northern, eastern, and southern suburbs were vulnerable.

Figure 3 shows the distributions of the traditional and healthy SVIs calculated by summing the score of each domain index, the integrated SVI calculated by adding the traditional and healthy SVIs, and the difference between the 2015 and 2019 SVIs. Different patterns were observed for the traditional and healthy SVIs. Based on the traditional SVI, the suburbs were more vulnerable than the central areas of the Capital region. The traditional SVI of 2019 was improved compared to 2015, especially in the southern area of the Capital region. However, there was an increase in vulnerability in some areas in the central, western, and eastern regions from 2015 to 2019. Based on the healthy SVI, the suburbs were more vulnerable than central areas of the Capital region. Differences in healthy SVI were found in the central areas above Seoul, whereas southern Seoul showed improvement. The overall vulnerability level improved in southern Seoul, central Gyeonggi, and the central-northern areas, but the central east areas of the Capital region were more vulnerable in 2019 than in 2015.

### Trend and spatial distribution of COVID-19 in the Capital region of South Korea

**Fig. 4:**
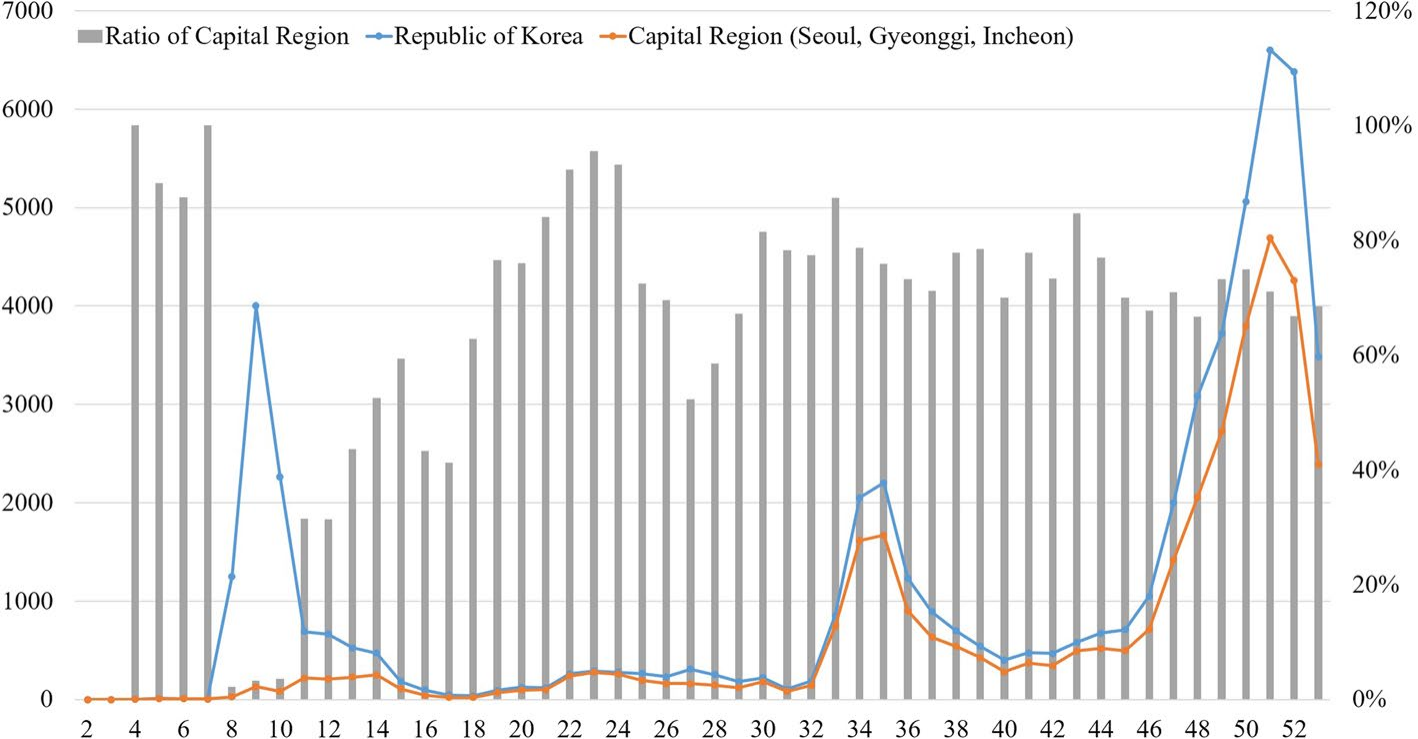
Confirmed COVID-19 Cases and their Proportions in the Capital Region. Note. Y-axis: Number of confirmed COVID-19 cases (left) and their proportion (in %) in the Capital Region (right) X-axis: week of 2020

Figure 4 shows the trend in the number of confirmed COVID-19 cases in the Capital region of South Korea in 2020. Following the first confirmed case in the country on January 19, 2020, the first, second, and third episodes of COVID-19 occurred in Weeks 8–11, 33–37, and 47–53 [56], respectively. A total of 62% of all confirmed COVID-19 cases in 2020 were in the Capital region, and 73% of all cases, excluding those from the first episode, were also in the Capital region.

**Fig. 5:**
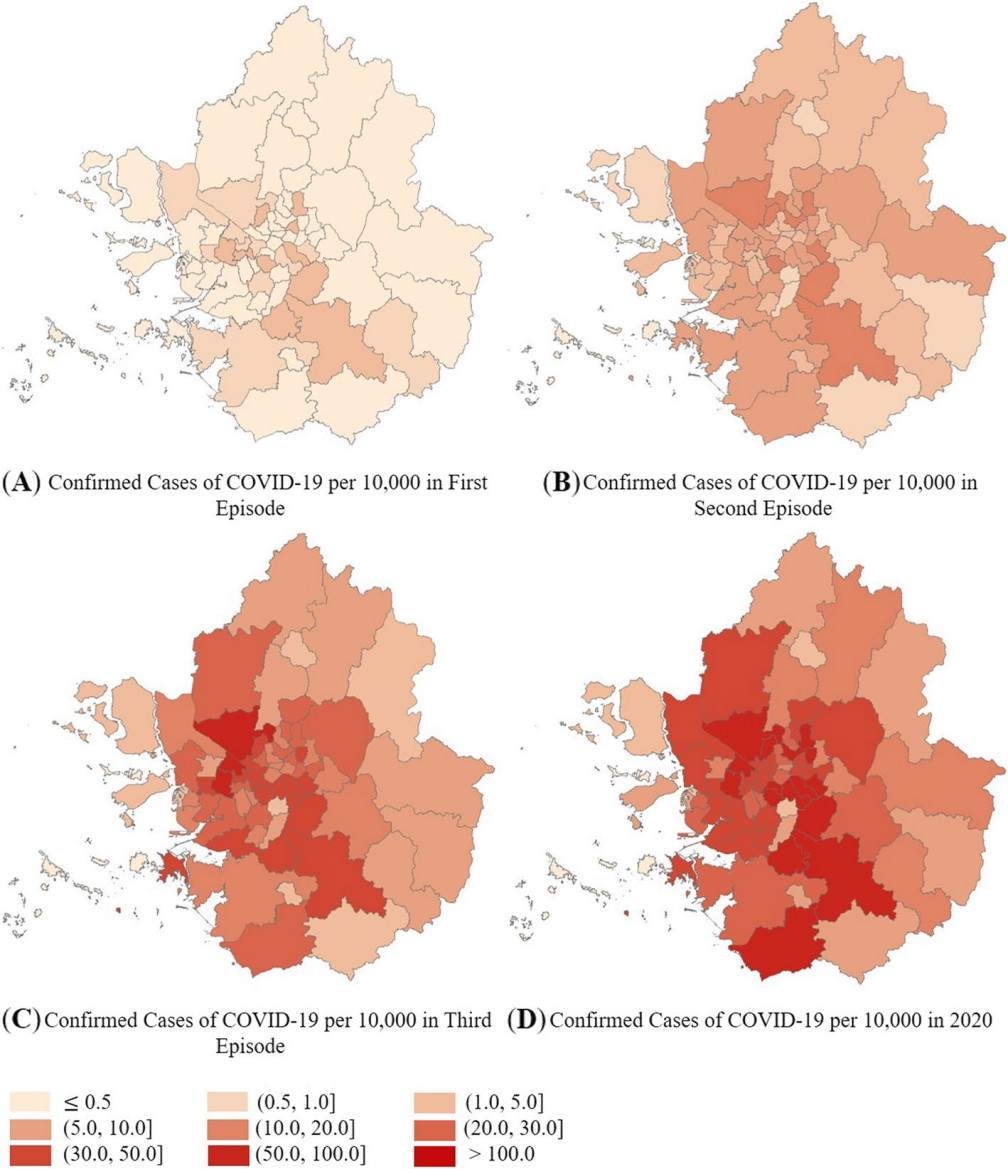
Spatial Distribution of Confirmed COVID-19 Cases per 10,000 Persons in the Capital Region. Note. Unit: Confirmed cases per 10,000 people

Figure 5 shows the spatial distribution of COVID-19 in the Capital region. Although the region consists of urban areas with similar socioeconomic characteristics, the COVID-19 cases were concentrated in certain areas. Since most of the cases were around Daegu Metropolitan City, a non-Capital region, the number of confirmed cases from the first episode in the Capital region was relatively low. Following the second episode, which started in the center of the Capital region, and the third episode, during which the number of confirmed cases increased drastically as a result of end-of-year gatherings and trips, COVID-19 spread across the Capital region. From the first episode, COVID-19 spread from the northwestern to the southern regions. The spread was relatively mild in the eastern and northern regions.

### Relationship between SVI and COVID-19

Table 2 shows the traditional, healthy, and integrated SVIs in 2015 and 2019, the change in the 2015 and 2019 SVIs, and their correlations with the number of confirmed COVID-19 cases in 2020. The traditional, healthy, and integrated SVIs in 2015 and 2019 were negatively correlated with the number of confirmed COVID-19 cases, implying that regions with high SVIs had fewer confirmed COVID-19 cases. The differences in the SVIs from 2015 to 2019 were not significantly correlated with the number of confirmed COVID-19 cases in the periods of three episodes or for the total period. However the correlation coefficient shown in Table 2 is based on simple linear correlation with no consideration of control variables, which means there is only a possible relationship between the two variables.

**Table 2:** Pearson’s Correlation between Confirmed COVID-19 cases and variables

	Confirmed COVID-19 Cases				Traditional SVI in 2015	Healthy SVI in 2015	Integrated SVI in 2015	Traditional SVI in 2019	Healthy SVI in 2019	Integrated SVI in 2019	Difference in Traditional SVI between 2015 and 2019	Difference in Healthy SVI between 2015 and 2019	Difference in Integrated SVI between 2015 and 2019
	First Episode	Second Episode	Third Episode	Total									
Traditional SVI in 2015	-0.3688 (0.0023)	-0.4660 (0.0001)	-0.4774 (0.0001)	-0.4798 (0.0000)	1								
Healthy SVI in 2015	-0.2659 (0.0309)	-0.4177 (0.0005)	-0.4251 (0.0004)	-0.4340 (0.0003)	0.3976 (0.0000)	1							
Integrated SVI in 2015	-0.3671 (0.0024)	-0.5206 (0.0000)	-0.5313 (0.0000)	-0.5387 (0.0000)	0.7707 (0.0000)	0.8911 (0.0000)	1						
Traditional SVI in 2019	-0.2387 (0.0536)	-0.3538 (0.0036)	-0.4225 (0.0004)	-0.4340 (0.0003)	0.2942 (0.0165)	0.5487 (0.0000)	0.5265 (0.0000)	1					
Healthy SVI in 2019	-0.3145 (0.0101)	-0.3038 (0.0132)	-0.4649 (0.0001)	-0.4348 (0.0003)	0.3380 (0.0055)	0.5148 (0.0000)	0.5247 (0.0000)	0.5732 (0.0000)	1				
Integrated SVI in 2019	-0.3108 (0.0111)	-0.3713 (0.0021)	-0.4997 (0.0000)	-0.4897 (0.0000)	0.3558 (0.0034)	0.5999 (0.0000)	0.5926 (0.0000)	0.8925 (0.0000)	0.8812 (0.0000)	1			
Difference in Traditional SVI between 2015 and 2019	-0.0062 (0.9606)	-0.0593 (0.6360)	-0.1200 (0.3370)	-0.1299 (0.2986)	-0.3317 (0.0065)	0.2943 (0.0165)	0.0403 (0.7479)	0.8041 (0.0000)	0.3556 (0.0034)	0.6596 (0.0000)	1		
Difference in Healthy SVI between 2015 and 2019	-0.0714 (0.5691)	0.0878 (0.4831)	-0.0744 (0.5527)	-0.0341 (0.7857)	-0.0322 (0.7976)	-0.4335 (0.0003)	-0.3169 (0.0095)	0.0678 (0.5885)	0.5494 (0.0000)	0.3416 (0.0050)	0.0870 (0.4875)	1	
Difference in Integrated SVI between 2015 and 2019	-0.0501 (0.6894)	0.0138 (0.9122)	-0.1334 (0.2856)	-0.1146 (0.3594)	-0.2576 (0.0368)	-0.0673 (0.5913)	-0.1741 (0.1620)	0.6179 (0.0000)	0.6058 (0.0000)	0.6900 (0.0000)	0.7701 (0.0000)	0.7024 (0.0000)	1

*Note*: Parentheses means *P*-value

Table 3 shows the results of multiple linear regression with control variables such as population in 2020. The two independent variables, the difference of the traditional SVI from 2015 to 2019 and the difference of healthy SVI from 2015 to 2019, were not correlated. Separate models were used for each episode. Models 1–3 were used to assess the relationship between SVI and the number of confirmed cases in the first, second, and third episodes. Model 4 was used to assess the relationship between SVI and the cumulative number of confirmed cases in 2020.

**Table 3:** Multiple linear regression analysis findings

	Model 1 (First Episode)		Model 2 (Second Episode)		Model 3 (Third Episode)		Model 4 (TOTAL)	
	$\beta$ *(t*-value*)*	Robust Standard Error	$\beta$ *(t*-value*)*	Standard Error	$\beta$ *(t*-value*)*	Standard Error	$\beta$ *(t*-value*)*	Standard Error
Constant	5.5525 (1.33)	4.1864	108.7861^***^ (3.07)	35.4267	489.4152^***^	114.0284	704.6469^***^ (4.23)	166.6855
Population	0.00002^***^ (4.84)	4.35e-06	0.0002^***^ (7.59)	0.00002	0.0006^***^ (8.34)	0.0007	0.0010^***^ (9.73)	0.0001
Integrated SVI in 2019	-1.0429^*^ (-1.75)	0.5974	-15.0633^**^ (-2.66)	5.6727	-68.8195^***^ (-3.77)	18.2588	-98.6967^***^ (-3.70)	26.6904
Difference in Traditional SVI between 2015 and 2019	3.6862^***^ (2.94)	1.2527	31.1475^***^ (3.78)	8.2454	113.8462^***^ (4.29)	26.5396	171.7925^***^ (4.43)	38.7952
Difference in Healthy SVI between 2015 and 2019	-0.6937 (-0.73)	0.9473	12.6517 (1.67)	7.5848	4.4486 (0.18)	24.4133	20.1448 (0.56)	35.6871
${R}^{2}$	0.527		0.629		0.698		0.743	
Adjusted ${R}^{2}$	0.496		0.605		0.678		0.726	
$F$-value	10.75^***^		25.84^***^		35.19^***^		44.11^***^	
Mean VIF	1.77		1.77		1.77		1.77	
White Test	33.89^***^		8.16		9.88		18.54	
Moran’s *I*	0.089		0.137		0.190		0.131	
N	66		66		66		66	

*Note: SVI* Social vulnerability index

^***^ *p* < 0.01, ﻿** *p *< 0.05, * *p *< 0.1

A significant positive correlation between the difference in the traditional SVI from 2015 to 2019 and the number of confirmed COVID-19 cases was observed in all models. An increase in any social vulnerability factor, such as income, age, socioeconomic disadvantage, housing, and environment was associated with an increase in the number of confirmed COVID-19 cases, although the magnitude of this association varied with the time of the episode. When the difference of the traditional SVI increased by one unit from 2015 to 2019, the number of confirmed COVID-19 cases increased to 171.7925 persons. However the difference in healthy SVI did not have a significant relationship with the number of confirmed COVID-19 cases for all models. The population had a significantly positive relationship with confirmed COVID-19 cases in all models, and the impact of population increased with the number of confirmed COVID-19 cases. The integrated SVI in 2019 had a significantly negative relationship with the confirmed COVID-19 cases in all models, indicating the low-vulnerability area of the integrated SVI had high confirmed cases of COVID-19. These results demonstrate that the increase of the traditional SVI’s difference has a positive relationship with the increasing number of confirmed COVID-19 cases at regional the level. The highly-vulnerable SVI, known to be positively correlated with a high number of confirmed COVID-19 cases as well as other diseases and disaster damage, had a negative relationship with confirmed COVID-19 cases in the Capital region of the South Korea.

## Discussion

Policies in South Korea for responding to COVID-19 have focused on social distancing and test-trace-isolate strategies, which are weaker than those adopted by some European nations and the United States [59]. Nevertheless, these strategies proved adequate in the early stages of the pandemic [60, 61]. However, despite the enforcement of a strengthened social distancing policy from July 2021 that bans private gatherings of four or more, events, and public gatherings, confirmed cases of COVID-19 have not decreased, but have rather proliferated. In particular, COVID-19 transmission has expanded around the Capital region. From December 1, 2021, the number of confirmed cases was 5,000 per day and 80% of them have been in the Capital region [62]. Apparently undaunted, the government has maintained a similar policy strategy to date.

Studies on the relationship between social vulnerability and COVID-19 using various SVIs are based on two notions. First, COVID-19 exhibits spatial heterogeneity despite widespread episodes [63–65]. Second, socioeconomic factors play a role in increasing the exposure and susceptibility of vulnerable groups to COVID-19 [17, 37, 63, 66]. The effects of SVIs in previous studies were varied. Many studies found positive relationships between COVID-19 cases, deaths, or incidence rate and SVI (15, 16, 23, 32, 33, 38]. Their studies used spatially-based analysis such as GIS and spatial regression models. Some studies suggest inconsistent relationships between COVID-19 and SVI. The relationships varied with the time of the outbreak or the variables that constructed SVIs or specific spatial conditions, such as rural areas [31, 40, 41]. Many studies focused on static SVI, not changes over time, and did not divide the characteristics of SVI into traditional vulnerability (e.g,. income, disadvantage, age) and healthy vulnerability (e.g. health infrastructure, prevention, health habitat).

This study focused on changes of SVI from 2015 to 2019, which was divided into traditional and healthy SVI. The analysis used the changes of the two SVIs with consideration of population and integrated SVI status in 2019 as control variables. The integrated SVI was found to be negatively correlated with the number of confirmed COVID-19 cases. However, the difference in traditional SVI from 2015 to 2019 (pre- pandemic) was positively correlated with the number of confirmed COVID-19 cases. The difference in healthy SVI from 2015 to 2019 was not correlated with the number of confirmed COVID-19 cases. Additionally, the impact of the difference in the traditional SVI on the number of confirmed COVID-19 cases varied by outbreak episode.

SVI, which is a potential factor for proactive decision-making by local governments [67], has an uneven distribution and impact. Episodic outbreaks of infection are highly unpredictable and can cause health and economic damage as they spread [68]; therefore, they must be controlled. While contact with a confirmed case is an important contributor to infection transmission [69], this study demonstrates that such transmission may also be associated with an increase in the traditional SVI, based on socioeconomic variables. A change in social vulnerability indicates how well a society can respond to or is prepared for a sudden disaster. The association between an increase in relative social vulnerability and the number of confirmed COVID-19 cases suggests that the outcomes of an episode can vary between regions with the same socioeconomic characteristics, depending on the local governments’ response and preparedness.

South Korea's response to COVID-19 was initially successful through rapid testing and contact tracing [51, 69, 70]. This response process is more strongly characterized by the central government-led crisis management response than that of the local government [71, 72]. This response was effective in 2020, but has limitations in 2021. In addition, it has been discussed that central government-led methods, such as in South Korea, should be careful in responding to a large population that is geographically spread [51]. Infectious diseases spread according to the regional hierarchy [73], which is demonstrated by the fact that COVID-19 spreads from major cities to small cities [74]. For sustainable management of infectious diseases, this means that local government-centered policies are needed rather than central government-led crisis management responses.

This study demonstrates the need for local governments to actively implement policies that can reduce the risk of infection transmission in regions where the traditional SVI has increased, even if they share the same socioeconomic characteristics. This means that isolation-and-quarantine, which is the most cost-effective intervention in the cluster outbreaks of COVID-19 [69], should be considered along with a personal protection policy for the area of increased social vulnerability. It is necessary to reduce risk of COVID-19 transmission by providing support to socially vulnerable classes who are inevitably exposed to risk in regions where vulnerability is higher compared to earlier SVIs.

One principal limitation of this study is that the findings do not show a causal relationship, only an exploratory relationship between social and health vulnerability and confirmed cases of COVID-19. Identification of the casual relationship would require data, including socioeconomic status, lifestyle, underlying diseases, and physical characteristics at the individual level with rigorous control treatment. However, the government has not yet released individual data due to privacy concerns in public health policy. Social vulnerability has an important relationship with case fatality rates; however, this data is not disclosed at the individual or regional level. Therefore, the relationships shown in this study should be interpreted as indicating potential risk. Further, the unit of analysis is a local administrative unit. To determine a more detailed relationship between social and health vulnerability indicators and confirmed COVID-19 cases, a more specific unit of analysis, such as community or census tract or spatial grid, is necessary. The local administrative unit used in this study reflects a broad range; consequently, there may be differences in traditional or healthy SVIs, despite being quoted in the same unit. This study focused on understanding the relationship between vulnerability and confirmed COVID-19 cases, using the local administrative unit as a representative spatial unit for differences in vulnerability. Given this understanding, over-interpretations should be avoided.

## Conclusion

This study examined the spatial distribution of SVIs and confirmed COVID-19 cases and analyzed the relationship between the two. The number of confirmed COVID-19 cases during the first, second, and third episodes and the cumulative number of confirmed cases in 2020 were analyzed. Suburbs were found to be highly vulnerable to COVID-19, based on the traditional SVI. Based on the healthy SVI, the northern regions were found to be highly vulnerable. The traditional SVI increased from 2015 to 2019 in some central, western, and eastern areas of the Capital region, whereas the healthy SVI increased in the central areas above Seoul. The number of confirmed COVID-19 cases was negatively correlated with the integrated SVI and positively correlated with an increase in the traditional SVI from 2015 to 2019. The magnitude of the impact of the traditional SVI on the number of confirmed COVID-19 cases increased with the time of the episode, controlling the population.

The results of this study have two implications for policy in regions where social vulnerability is increasing to control COVID-19 transmission. First, the government needs to focus its attention on regions where social vulnerability has increased as compared to that in the past, while providing subsidies. The present results imply that the relationship between the traditional SVI and socioeconomic status should be considered, as opposed to the relationship between healthy SVI and infectious diseases, even in cases of infectious diseases with high uncertainty. Rather than providing equal subsidies to all individuals to respond to infectious diseases, this study suggests that preferential payment should be made to regions in which social vulnerability may have increased at the local level. Second, this study indicated the need for continuous government monitoring and management of the traditional SVI in relation to regional socioeconomic status. The SVI is not static; it is relative and changes over time. Damage can be prevented when SVI-deteriorated and SVI-improved areas are continuously monitored in the light of various risks as compared to the past. 

## Supplementary Information

**Additional file 1.**

## Data Availability

Publicly available datasets were analyzed in this study. The data used in Table 1 are available from the webpage of Korea Statistical Information Service by Statistics Korea (https://kosis.kr/index/index.do). The data about COVID-19 are available from the official webpage of the Korea Disease Control and Prevention Agency (http://ncov.mohw.go.kr/en/tcmBoardView.do?brdId=12&brdGubun=125&dataGubun=&ncvContSeq=4552&contSeq=4552&board_id=&gubun =).

## Abbreviations

COVID-19: Coronavirus disease 2019
GIS: Geographic information system
SVI: Social vulnerability index
US CDC: Center for disease control and prevention in the United States
