# Supplementary material for: Exploring spatial distribution of social vulnerability and its relationship with the Coronavirus disease 2019: the Capital region of South Korea
Source: BMC Public Health. 2022 Oct 10;22:1883. doi: 10.1186/s12889-022-14212-7 (PMC9548431; doi:10.1186/s12889-022-14212-7)
Supplement: Supplementary file 1 — Additional file 1. [file 12889_2022_14212_MOESM1_ESM.docx]

# Appendix

Table A1. Descriptive statistics

| Variable | | Mean | Standard Deviation | Min | Max |
| --- | --- | --- | --- | --- | --- |
| Confirmed COVID-19 Cases | First Episode  (Model 1) | 6.909 | 7.637 | 0 | 35 |
|  | Second Episode  (Model 2) | 84.364 | 60.091 | 0 | 262 |
|  | Third Episode  (Model 3) | 323.152 | 214.294 | 0 | 968 |
|  | Total  (Model 4) | 525.106 | 339.795 | 0 | 1443 |
| Population | | 394519.8 | 256254.8 | 20455 | 1186078 |
| Traditional SVI in 2015 | | 2.971 | 0.480 | 2.110 | 4.194 |
| Healthy SVI in 2015 | | 2.477 | 0.674 | 0.722 | 4.077 |
| Integrated SVI in 2015 | | 5.448 | 0.970 | 3.980 | 7.836 |
| Traditional SVI in 2019 | | 2.496 | 0.761 | 0.897 | 4.269 |
| Healthy SVI in 2019 | | 2.492 | 0.727 | 0.848 | 3.965 |
| Integrated SVI in 2019 | | 4.988 | 1.320 | 2.491 | 8.119 |
| Difference in Traditional SVI between 2015 and 2019 | | -0.474 | 0.771 | -1.976 | 1.239 |
| Difference in Healthy SVI between 2015 and 2019 | | 0.015 | 0.691 | -1.628 | 1.501 |
| Difference in Integrated SVI between 2015 and 2019 | | -0.459 | 1.079 | -2.651 | 2.118 |

Table A2. Summary of variables in SVIs

| Tradtional SVI | Domain | Variable | 2015 | | | | 2019 | | | |
| --- | --- | --- | --- | --- | --- | --- | --- | --- | --- | --- |
|  |  |  | Mean | Standard Deviation | Min | Max | Mean | Standard Deviation | Min | Max |
|  | A.Age | Older population | 0.126 | 0.038 | 0.074 | 0.286 | 0.150 | 0.042 | 0.085 | 0.317 |
|  | B. Socioeconomic disadvantage | Foreign minorities | 0.555 | 0.188 | 0.170 | 0.895 | 0.617 | 0.186 | 0.209 | 0.936 |
|  |  | Vulnerable groups | 0.033 | 0.021 | 0.007 | 0.112 | 0.038 | 0.023 | 0.009 | 0.091 |
|  |  | Disabled persons | 0.045 | 0.012 | 0.025 | 0.085 | 0.047 | 0.013 | 0.025 | 0.086 |
|  | C. Housing | Old houses | 0.132 | 0.100 | 0.015 | 0.603 | 0.156 | 0.084 | 0.014 | 0.424 |
|  | D. Income | Pension income | 3972.157 | 462.059 | 3163.280 | 5744.391 | 4613.129 | 502.795 | 3790.157 | 6485.335 |
|  |  | Earned income | 34.016 | 8.002 | 23.677 | 63.203 | 38.248 | 9.074 | 27.753 | 71.198 |
|  | E. Environment | Particulate Matter (PM) 2.5 | 25.525 | 2.652 | 19.917 | 32.708 | 24.804 | 2.502 | 18.833 | 31.688 |
|  |  | Particulate Matter (PM)10 | 49.840 | 5.357 | 42.417 | 65.833 | 43.604 | 4.200 | 32.250 | 55.000 |
| Healthy SVI | F. Prevention | Rate of engaging in physical activities | 22.585 | 4.067 | 15.100 | 35.700 | 23.596 | 3.632 | 16.200 | 31.600 |
|  |  | Influenza immunization rate | 34.545 | 3.355 | 28.400 | 46.500 | 41.446 | 4.098 | 32.700 | 56.100 |
|  | G. Health-related habits | Smoking | 21.959 | 2.784 | 11.600 | 28.400 | 19.670 | 3.387 | 11.800 | 28.800 |
|  |  | Obesity | 26.048 | 2.358 | 21.400 | 32.200 | 33.402 | 3.748 | 24.400 | 44.800 |
|  | H. Disease | Hypertension | 20.118 | 1.996 | 15.100 | 24.300 | 20.049 | 2.166 | 15.000 | 27.400 |
|  |  | Diabetes | 7.833 | 1.208 | 4.500 | 10.200 | 8.104 | 1.411 | 5.000 | 11.000 |
|  | I. Healthcare infrastructures | Number of medical facilities | 17.592 | 8.043 | 10.087 | 57.480 | 18.926 | 8.812 | 10.736 | 59.626 |
|  |  | Number of beds | 56.096 | 35.391 | 0.037 | 187.196 | 55.436 | 34.959 | 0.044 | 158.435 |
|  |  | Number of professionals | 15.361 | 12.819 | 4.985 | 83.993 | 17.639 | 14.324 | 6.530 | 92.167 |
|  | J. Mortality | Mortality of respiratory diseases | 43.146 | 17.904 | 23.000 | 115.600 | 60.823 | 25.096 | 33.700 | 159.800 |
|  |  | Mortality of infectious and parasitic diseases | 12.355 | 5.677 | 5.000 | 37.800 | 13.862 | 5.409 | 5.800 | 33.900 |
